# How to Be Young at Eighty

**Published:** 1883-05

**Authors:** 


### HOW TO BE YOUNG AT EIGHTY.

ONE great reason why I never had a really sick day in my life was that I was born and nurtured in a sweet little home, where we lived on oatmeal and milk, and brown bread with butter once a week, potatoes and a bit of meat when we could catch it, and then oatmeal again. So I don't know to-day as I have a system or a constitution or a digestion at all; I am never conscious of such a thing. Hence I say we must go back to the father and mother for the first answer to our question. Thousands of young men come to such cities as this from the Green Mountains, or from New Hampshire, or Maine, with just such a constitution as mine. They have within them all the conditions for a long, sweet life. They can use their years wisely and well, and write at the end of each one "Value received." Or they can overdraw the account, as many do, God help them! Instead of saying at fifty, "I am young yet," they will say at forty, "I am old indeed." They are so ambitious to get on, some of them, that they use up two days in one and waste their vital powers. They ride when they ought to walk down town, and they take "a little something," as they say, to restore their lax energies, for which they have to chew a clove or a coffee berry, I am told. They are overdrawing their account, I say, and some day nature and the grace of God will shut down on them. Those who do differently keep a good digestion, stay young and buoyant, love good, sweet company, and are not ashamed to look their mothers and sisters in the eye or kiss them. Another secret that must be known to be young at eighty is that you must keep faith in the common manhood and womanhood and in the advancing progress of the day. Never say that the past is better than to-day is; read the new books, understand all the new ideas; and keep your faith in God and man and in the victory of good over evil.—*Dr. Collyer.*

